# Exploring sensory, motor, and pain responses as potential side or therapeutic effects of sub-2 mA, 400 Hz transcranial pulsed current stimulation

**DOI:** 10.1371/journal.pone.0290137

**Published:** 2023-12-13

**Authors:** Shapour Jaberzadeh, Maryam Zoghi

**Affiliations:** 1 Department of Physiotherapy, Monash Neuromodulation Research Unit, Monash University, Melbourne, Victoria, Australia; 2 Discipline of Physiotherapy, Federation University, Churchill, Victoria, Australia; Federal University of Paraiba, BRAZIL

## Abstract

**Background:**

Various brain stimulation devices capable of generating high-frequency currents are readily available. However, our comprehension of the potential side or therapeutic effects associated with high-frequency transcranial pulsed current stimulation (tPCS), particularly concerning the new 400 Hz tPCS device, AscenZ-IV Stimulator, developed by AscenZion Neuromodulation Co. Pte. Ltd. in Singapore, remains incomplete.

**Objective:**

This study examines preliminary parameters for the safe and comfortable application of 400 Hz tPCS at intensities below 2 mA.

**Methods:**

In a cross-sectional study, 45 healthy participants underwent sub-2 mA 400 Hz tPCS to assess sensory, motor, and pain thresholds on the dominant side. Study 1 (N = 15) targeted the primary motor cortex of the right-hand area, while study 2 (N = 30) focused on the back of the right forearm.

**Results:**

Study one showed that increasing the current intensity gradually resulted in no responses at sub-0.3 mA levels, but higher intensities (p < 0.001) induced sensory perception and pain responses. Study two replicated these findings and additionally induced motor responses along with the sensory and pain responses.

**Conclusion:**

Despite the theoretical classification of tPCS as a subsensory level of stimulation, and the expectation that individuals receiving this type of current should not typically feel its application on the body, this high-frequency tPCS device generates different levels of stimulation due to the physiological phenomenon known as temporal summation. These novel levels of stimulation could be viewed as either potential “side-effects” of high frequency tPCS or as additional “therapeutic benefits”. This dual capacity may position the device as one that generates both neuromodulatory and neurostimulatory currents. Comprehensive comprehension of this is vital for the development of therapeutic protocols that incorporate high-frequency tPCS.

## Introduction

Transcranial electrical stimulation methods, such as transcranial Pulsed Current Stimulation (tPCS), commonly utilize intensities below 2 mA [1]. These intensities fall into the subsensory range [2]. They do not provoke noticeable sensory, motor, or painful reactions, rendering recipients unable to perceive the current [2].

TPCS is a versatile neuromodulation technique effective across a wide frequency range, spanning from direct current (DC) to 5 kHz [3]. The existing literature on pulsed currents suggests that at low intensities and frequencies, currents do not trigger any sensory or motor responses [4]. However, this current at higher frequencies (between 30–80 Hz) can activate sensory fibers in the skin, resulting in diverse sensations. At higher frequencies (>80 Hz) it has the potential to produce stronger sensations by engaging sensory, motor, and pain fibers [4]. During application of this type of current, these effects may have a dual nature, with the potential to generate both undesired side effects or novel therapeutic effects. Unfortunately, limited literature exists on participant experiences with different frequency ranges. This limitation impedes our understanding of potential risks and effects.

In recent years, several companies have developed devices capable of generating tPCS at various frequencies. These devices have become valuable tools in neuroscience research and clinical settings. Noteworthy devices include the NeuroConn DC Stimulator Plus, Soterix Medical 1x1 tES Device, Brainbox Neurostimulation System, and Starstim. Among them, the AscenZ-IV Stimulator stands out as the sole device designed specifically for high-frequency tPCS for home use [5]. With a fixed frequency of 400 Hz and Class B approval in Singapore, it enables patients to self-administer tPCS. Although currently limited to Singapore, the introduction of the AscenZ-IV Stimulator marks a significant advancement in patient-centric healthcare. The decision to utilize 400 Hz tPCS draws inspiration from theoretical models proposing the role of high-frequency brain activities in signal transmission and the identification of cortical circuits in the primary motor cortex [6–8]. Furthermore, 400 Hz stimulation has demonstrated the ability to enhance the detection of rapid changes in ultra-fast EEG activity (60–1000 Hz). It has also been observed to impact sensory-motor control in healthy individuals [9].

However, the potential implications of using high-frequency tPCS are not yet fully understood. Further research is necessary to determine the safety and efficacy of these higher frequencies, as well as the optimal parameters for different applications. While the development of neuromodulatory devices capable of producing high-frequency tPCS holds promise, thorough investigation is required. This investigation aims to comprehend the potential benefits and risks associated with their utilization.

**Fig 1:**
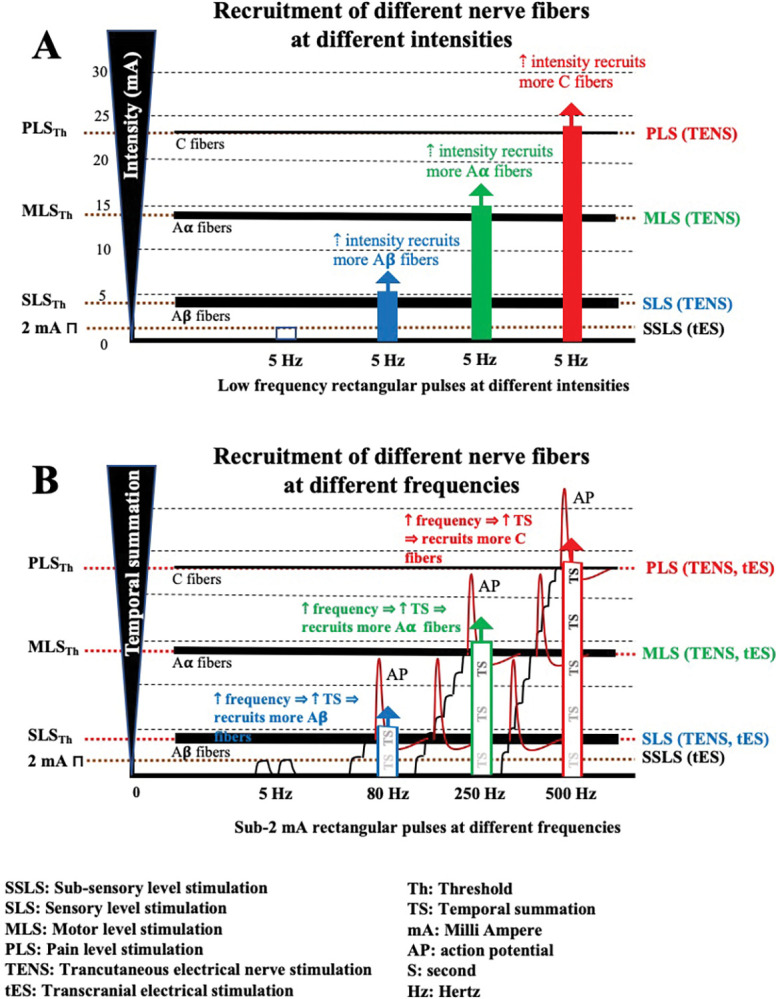
A) The effects of electrical stimulation vary based on intensity levels. Sub-sensory stimulation, commonly used in tES techniques, occurs at low intensities. Increasing the intensity recruits different nerve fibers, leading to sensory, motor, and pain responses. B) The effects of electrical stimulation also vary based on frequency levels. Intensity levels below 2 mA result in sub-sensory stimulation level, particularly at low frequencies below 10–20 Hz. Increasing the frequency, causes increase in temporal summation and recruits different nerve fibers, resulting in sensory, motor, and pain levels of stimulation. Note: Fig 1 is not based on empirical data; rather, it has been constructed using established neurophysiological principles and concepts.

To effectively address the problem at hand, it is necessary to consider different levels of electrical stimulation. The intensity of the current can be adjusted to achieve specific outcomes. Subsensory level stimulation (SSLS) [10] involves low-intensity electrical stimulation and is used in various therapeutic techniques, such as tissue repair and transcranial electrical stimulation [11–13]. These techniques aim to modulate cortical neuron activity and have shown promise in treating neurological and psychiatric conditions. As the current intensity increases, sensory fibers are the first to be recruited, resulting in Sensory level stimulation (SLS, Fig 1A) [14,15]. SLS is primarily employed for pain treatment [16,17]. Further increasing the intensity stimulates motor nerve fibers with higher activation thresholds, leading to Motor level stimulation (MLS, Fig 1A) [15]. MLS is used to stimulate muscles, enhancing strength and control of pain [18]. At higher intensities, pain fibers with the highest activation threshold are recruited, known as the Pain level of stimulation (PLS, Fig 1A) [19]. PLS is primarily used for pain treatment and diagnostic purposes [16,17].

The optimal current intensity range for tPCS and other tES techniques (e.g., transcranial direct current stimulation (tDCS), transcranial alternating current stimulation (tACS) or transcranial random noise stimulation (tRNS) is typically between 0.1–2.0 mA [20–22]. This falls under SSLS (Fig 1A and 1B), meaning it remains below the activation threshold for sensory, motor, or pain neurons [4].

Adhering to the SSLS range is crucial for safe and effective tPCS treatments. By staying within this range, practitioners can achieve targeted neural modulation while minimizing non-specific effects, offering therapeutic possibilities for neurological and psychiatric conditions by modulating cortical excitability. However, with frequency-based tES techniques like tPCS or tACS, higher frequencies pose a challenge. The effects of intensities below 2 mA at high frequencies can change significantly. At these frequencies, temporal summation occurs [23], leading to increased stimulation levels through the recruitment of sensory, motor, and pain fibers (Fig 1B) [24,25]. This dual-edged characteristic can result in both undesired side effects such as intense sensations, muscle contractions, or even pain. Conversely, it can also lead to desired therapeutic effects and open new horizons for the induction of therapeutic benefits. The finding in this research could potentially be extended to include other tES methods, including tACS and tRNS. These findings may serve as a foundational basis for understanding the broader applicability of tES techniques and their potential effects on neural modulation. Such extrapolation could significantly contribute to our comprehension of the broader field of neuromodulation and its therapeutic applications.

The main objective of this study is to examines preliminary parameters for the safe and comfortable applications of 400 Hz tPCS at intensities below 2 mA. We hypothesize that administering 400 Hz tPCS at intensities below 2 mA, will result in induction of sensory, motor, and pain responses.

## Materials and methods

The study approved by Monash University’s human ethics committee and conducted (between 1 March-30 June 2023) at the Neuromodulation Research Unit, included 45 healthy participants (24 females, 21 males, ages 18–55).

Eligible participants for this study met specific criteria: they maintained a normal body mass index within the range of 18.5–25 kg/m^2^ [26], exhibited overall good health without any neurological or psychiatric disorders, and had no significant history of brain injuries or seizure disorders. Additionally, they were not currently taking medications that could potentially influence brain function or disrupt the study, and pregnancy was an exclusion criterion. All enrolled individuals were required to be right-handed [27] and possess sufficient language proficiency to comprehend and adhere to provided instructions. Exclusion criteria [28–32] extended to the presence of metal implants in the cranial or cervical regions [33], the existence of indwelling bladder or heart stimulators within the body, underlying medical conditions that contraindicated the application of pulsed currents over the scalp and peripherally over the skin, as well as sensory impairments that might impact their ability to perform study-related tasks. These rigorous criteria were meticulously applied to ensure participant homogeneity and the integrity of research outcomes.

**Fig 2:**
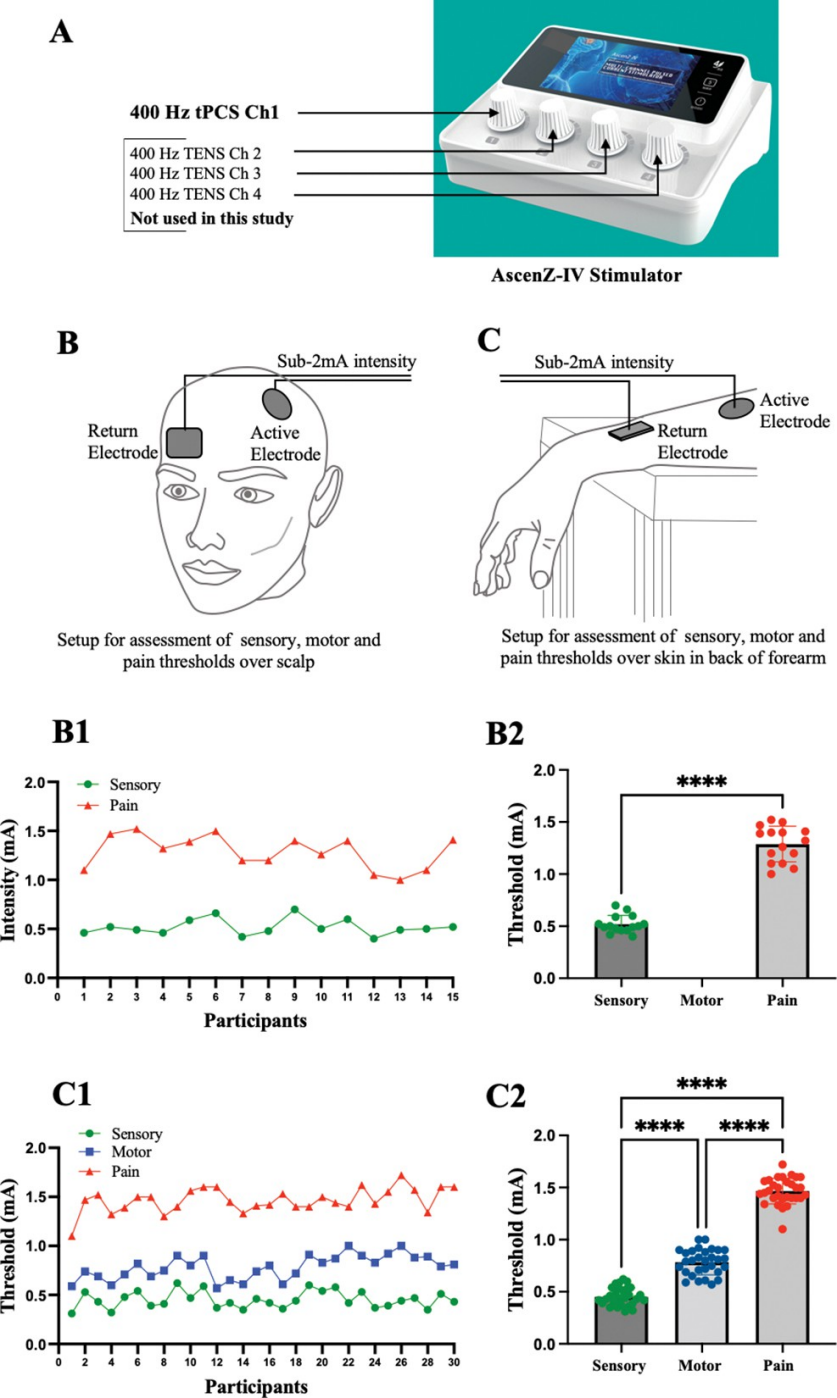
Four channel AscenZ-IV Stimulator. Only channel 1 is used in this study (A); Setup for assessment of sensory, motor and pain thresholds over scalp (B); and over dorsum of forearm (C); Elicited sensory (Green), and pain (Red) responses in 15 healthy participants using sub-2 mA intensities (B1); the pairwise comparisons of the means for the different levels of stimulation (B2); Elicited sensory (Green), motor (Blue) and pain (Red) responses in 30 healthy participants using sub-2 mA intensities (C1); and the pairwise comparisons of the means for the different levels of stimulation (C2). **** indicates significant difference with p<0.001. Image 1 in this figure republished from AscenZion website under a CC BY license, with permission from Sandra Zhong, CEO, AscenZion Neuromodulation Co. Pte Ltd Sandra Zhong, CEO, AscenZion Neuromodulation Co. Pte Ltd, original copyright 2023.

We obtained written informed consent through the signing of a consent form (witnessed by the investigators) adhering to the principles outlined in the Declaration of Helsinki. Data collection involved the use of the AscenZ-IV Stimulator to administer 400 Hz tPCS at an intensity of below 2 mA (Fig 2A).

In study 1 (n = 15) a 5 cm circular electrode positioned on the left primary cortex of the hand area (C3, 10–20 EEG system), and a 6x4 cm rectangular electrode on the contralateral forehead (Fig 2B). The primary dependent variables in this study include the thresholds for elicitation of "sensory" and "pain" responses, all measured in units of "mA". Sensory perception is characterized by the recognition of a "tingling" sensation [34]. Pain perception is described as the transformation of a tingling sensation into a painful experience [35,36]. This choice was made because there were no muscles situated beneath the electrode over scalp, and as a result, we were unable to provoke any motor responses.

In Study 2 (n = 30), two 4x6 cm electrodes were placed over the extensor muscles of the wrist and fingers of the dominant limb (Fig 2C). In this study, "sensory," "motor," and "pain" thresholds were selected due to their grounding in the physiological responses of the human body to electrical pulses at various intensities. Sensory perception is characterized by the recognition of a "tingling" sensation. Motor responses are identified as the initiation of muscle contractions at the stimulated site [37]. Pain perception is characterized by the transition from a tingling sensation to an unpleasant or painful experience [35,36].

During experiments, volunteers acclimated for 15 minutes in a controlled environment [38]. The primary dependent variables in this study include the thresholds for elicitation of "sensory," "motor," and "pain" responses, all measured in units of "mA". Current intensity gradually increased from 0 mA, and the participants were then instructed to report their first sensation as the current continued to increase [39]. The sensory perception threshold, indicating the minimum current to activate sensory fibers, was determined. Then the motor threshold, representing the intensity for minimal detectable muscle contraction was determined. Finally, the pain threshold was identified as the transition from sensory perception to unpleasant or painful sensation. Participants were informed that the pain threshold corresponded to a level of 1–2 on the 0−10 VAS scale.

Study 1 employed GraphPad Prism version 9.3.1 for Windows, GraphPad Software, Boston, Massachusetts USA, www.graphpad.com. In study 1, we utilized a Paired t-test (α = 0.05) to analyze two datasets obtained from the assessment of "sensory" and "pain" stimulation levels, as illustrated in Fig 2 B2.

In study 2, the same software was used, conducting a one-way ANOVA followed by the Tukey test (α = 0.05). Results are presented in Fig 2 C2.

## Results

Both studies employed 400 Hz tPCS at intensities below 2 mA, delivered using the AscenZ-IV Stimulator (Fig 2A).

The study 1 comprised a diverse participant group (N = 15), characterized by a broad range of demographic attributes. The age of the participants spanned from 18 to 55 years, with a mean age of 32.5 years (± 6.2 SD). In terms of gender distribution, the sample was nearly evenly split, with 8 identifying as male and 7 as female. Participant weights ranged from 48 to 85 kg, with an average weight of 62.5 kg (± 9.3 SD). The BMI of the participants ranged from 20.6–24.4, with an average BMI of 22.6 kg (± 2.2 SD).

In study 1, we administered stimulation over the scalp, specifically targeting the M1 region responsible for hand functions. Consequently, this application primarily elicited sensory and pain perceptions, as there were no muscles directly beneath the active electrode over M1, resulting in the absence of detectable motor responses. Therefore, in this study, stimulation evoked responses at two distinct sensory and pain levels (Fig 2, B1 and B2). Significantly different thresholds were observed, with the sensory threshold (0.519 mA) consistently lower than the pain threshold (1.288 mA) (p<0.001, t = 21.66, df = 14).

The study 2 comprised a diverse participant group (N = 30), characterized by a broad range of demographic attributes. The age of the participants spanned from 19 to 53 years, with a mean age of 34.1 years (± 8.1 SD). In terms of gender distribution, 14 of participants were male and 16 as female. Participant weights ranged from 47 to 89 kg, with an average weight of 64.5 kg (± 7.7 SD). The BMI of the participants ranged from 20.2–24.8, with an average BMI of 21.9 kg (± 2.2 SD).

In study 2, we employed stimulation on the back of the forearm, with the active electrode positioned over the skin covering the wrist extensor muscles. As a result, this application not only induced sensory and pain perceptions but also triggered the contraction of the wrist extensor muscles, leading to observable motor responses as muscle contractions.

The stimulation protocol evoked responses at three distinct sensory, motor, and pain levels. Significant differences were observed among these levels, with the sensory threshold (0.4510 mA) consistently lower than the motor (0.7840 mA) and pain (1.466 mA) thresholds (p<0.001, F(2, 87) = 641.4). Lower and upper 95% confidence interval (CI) of means are 0.419 and 0.483 for sensory, 0.738 and 0.829 for motor and 1.419 and 1.512 for pain responses. These findings emphasize the diverse effects and thresholds associated with each level of stimulation.

## Discussion

The study findings show that 400 Hz tPCS at intensities below 2 mA applied over the scalp (study 1) induces sensory and pain stimulation levels, while applying this current over the back of the forearm induces sensory, motor, and pain stimulation levels (study 2). Remarkably, all participants exhibited high tolerance to the stimulation effects in both studies.

Our hypothesis was that 400 Hz tPCS at intensities below 2 mA would induce significant sensory, motor, and pain responses. The study findings support this hypothesis. However, lack of comparable studies in this area, preventing direct comparisons with existing literature. Nonetheless, our results align with extensive research on higher stimulation frequencies, demonstrating that increased frequencies enhance temporal summation and produce stronger effects. This study addresses an important gap by investigating 400 Hz tPCS at intensities below 2 mA, contributing to understanding of how subsensory level stimulations at low frequencies become a current sufficient for induction of sensory, motor or pain levels of stimulation. Further research is needed to uncover underlying mechanisms and establish clinical implications, expanding knowledge of tPCS as a potential therapy.

How 400 Hz tPCS at intensities below 2 mA induces sensory, motor, and pain responses is a key question. Subsensory level stimulations can gain sufficient strength, particularly at higher frequencies, to elicit sensory, motor, or pain responses through a phenomenon termed temporal summation [40]. Temporal summation takes place when multiple weak electrical pulses are rapidly delivered, effectively aggregating their effects over time. At subsensory levels, these individual pulses may lack the strength to elicit noticeable responses independently [2]. However, when these pulses are rapidly administered at higher frequencies, they accumulate and surpass the threshold for activating sensory, motor, or pain fibers and therefore perception of sensory stimuli, muscle contractions (motor responses), or the sensation of pain [2]. It’s important to highlight that motor fibers exhibit a higher threshold in comparison to sensory fibers [41], whereas pain fibers possess an even higher threshold than motor fibers.

Another key question is how high-frequency applied currents affect tissue impedance and therefore current transmission through the scalp and cranium. Frequency influences tissue impedance and current transmission [25]. Tissue impedance represents the resistance encountered by electric current in tissues [42]. Higher frequency currents encounter less impedance, enabling deeper penetration into tissue and activation of more neurons or muscle fibers [43]. Understanding the frequency-tissue impedance relationship is critical for designing effective tPCS protocols for treating pathological conditions.

The study discovered a surprising aspect of 400 Hz tPCS at intensities below 2 mA that surpasses common knowledge. It was found that this type of stimulation can be classified not only as a neuromodulatory current but also as a neurostimulatory current. An important question is how this type of stimulation works within the range that’s known to be helpful for therapy. Based on the results of this study, it appears that 400 Hz tPCS can serve as a neuromodulation technique when used at intensities lower than 0.5 mA. At these lower intensities, the stimulation is considered to be below the threshold of sensory perception. On the other hand, it can also be employed at higher intensities, specifically between 0.5 and 2 mA, where it has stimulatory effects on the scalp. These higher stimulatory intensities may lead to more significant impacts on the neurons within the brain cortex. To better understand its stimulating effects on cortical neurons, additional studies are necessary, particularly examining its impact on corticospinal excitability using transcranial magnetic stimulation. Furthermore, more research is needed to fully comprehend the clinical implications of this phenomenon.

The current study has a number of limitations including its focus solely on 400 Hz tPCS, limited generalizability to older adults due to a predominantly young sample, and the need for research on individuals with pathological conditions. Future research, exploring the impacts of different frequencies, intensities, pulse widths, inter-pulse intervals, and duty cycles, as well as the creation of a comprehensive reference table, carries substantial potential for providing clear and practical guidelines for clinical use of tPCS. In addition, future studies on older adults and patients with pathologies, and considering gender-specific effects in future studies will enhance our understanding of tPCS effects.

## Conclusions

The findings highlight that sub-2 mA 400 Hz tPCS, recruiting sensory, motor, and pain fibers through temporal summation, presents both further side and therapeutic effects. To ensure safe clinical use, it is important to minimize unwanted sensations, movements, and pain. Understanding the mechanisms and effects is crucial for evaluating therapeutic potential and ensuring safe application.

During the preparation of this work the author(s) used Chat GPT in order to improve language and readability. After using this tool/service, the author(s) reviewed and edited the content as needed and take(s) full responsibility for the content of the publication.

## Supporting information

S1 File Threshold data recorded during scalp stimulation. (TXT)

S2 File Threshold data recorded during wrist extensor stimulation. (TXT)
